# Stable and selective permeable hydrogel microcapsules for high-throughput cell cultivation and enzymatic analysis

**DOI:** 10.1186/s12934-020-01427-9

**Published:** 2020-08-27

**Authors:** Salvatore Di Girolamo, Chasper Puorger, Georg Lipps

**Affiliations:** University of Applied Sciences and Arts Northwestern Switzerland, Institute for Chemistry and Bioanalytics, Hofackerstrasse 30, 4132 Muttenz, Switzerland

**Keywords:** Compartmentalization, High-throughput enzyme screening, Hydrogels, Layer-by-layer technology, Transpeptidation, Sortases, COPAS, MTT assay, Caged Biocatalyst

## Abstract

**Background:**

Miniaturization of biochemical reaction volumes within artificial microcompartments has been the key driver for directed evolution of several catalysts in the past two decades. Typically, single cells are co-compartmentalized within water-in-oil emulsion droplets with a fluorogenic substrate whose conversion allows identification of catalysts with improved performance. However, emulsion droplet-based technologies prevent cell proliferation to high density and preclude the feasibility of biochemical reactions that require the exchange of small molecule substrates. Here, we report on the development of a high-throughput screening method that addresses these shortcomings and that relies on a novel selective permeable polymer hydrogel microcapsule.

**Results:**

Hollow-core polyelectrolyte-coated chitosan alginate microcapsules (HC-PCAMs) with selective permeability were successfully constructed by jet break-up and layer-by-layer (LBL) technology. We showed that HC-PCAMs serve as miniaturized vessels for single cell encapsulation, enabling cell growth to high density and cell lysis to generate monoclonal cell lysate compartments suitable for high-throughput analysis using a large particle sorter (COPAS). The feasibility of using HC-PCAMs as reaction compartments which exchange small molecule substrates was demonstrated using the transpeptidation reaction catalyzed by the bond-forming enzyme sortase F from *P. acnes*. The polyelectrolyte shell surrounding microcapsules allowed a fluorescently labelled peptide substrate to enter the microcapsule and take part in the transpeptidation reaction catalyzed by the intracellularly expressed sortase enzyme retained within the capsule upon cell lysis. The specific retention of fluorescent transpeptidation products inside microcapsules enabled the sortase activity to be linked with a fluorescent readout and allowed clear separation of microcapsules expressing the wild type SrtF from those expressing the inactive variant.

**Conclusion:**

A novel polymer hydrogel microcapsule-based method, which allows for high-throughput analysis based on encapsulation of single cells has been developed. The method has been validated for the transpeptidation activity of sortase enzymes and represents a powerful tool for screening of libraries of sortases, other bond-forming enzymes, as well as of binding affinities in directed evolution experiments. Moreover, selective permeable microcapsules encapsulating microcolonies provide a new and efficient means for preparing novel caged biocatalyst and biosensor agents.

## Background

The availability of technologies for spatial separation and processing of a large number of cellular clones is of utmost importance in pharmaceutical and biotechnological research [1–3]. The goal of such technologies is to perform laboratory assays in a highly parallel fashion and at miniaturized scale so that clones from a population can be studied with high-throughput screening methods. Over the last two decades, several powerful strategies have been developed that allowed for high-throughput detection, analysis, sequencing and directed evolution of cells and their components [4–9].

In the most widely used approach, clones from a population are compartmentalized in the wells of multi-well plates and each clone is individually analyzed using sophisticated liquid handling robot-stations. However efficient, this approach requires huge investments in terms of money, time and space, and imposes a throughput limit of ~ 10^5^ variants per screen [10]. Over recent years, technologies that rely on man-made microcompartments have emerged as a valid alternative to conventional high-throughput analysis methods. Microcompartment-based technologies enable the further miniaturization of laboratory assays and provide significantly higher throughputs, which in turn permits faster exploration of higher library diversity [11]. Microcompartments provide the perfect platform for evolutional studies: they enable high-throughput screening of spatially separated clones, while maintaining the linkage between the phenotype (the optical readout that originates from catalysis) and the genotype (the nucleic acid sequence that encodes the catalysts) [12]. This is an essential requirement for any directed evolution strategy.

Since the pioneering work by Tawfik and Griffiths [13], who first introduced the term in vitro compartmentalization (IVC), screening platforms that rely on single, double and microfluidic-based emulsion droplets as compartments have been successfully developed for several catalytic activities including polymerases [14], hydrolases [15, 16], oxidoreductases [17, 18], lyases [19] or transferases [20].

In a typical experiment, protein catalysts produced by a single cell or via in vitro translation are co-compartmentalized within an emulsion droplet with a fluorogenic substrate. Substrate conversion to a fluorescent product allows identification of catalysts with improved properties based on fluorescence readout. In this process, the droplet boundary has the fundamental role of retaining the fluorescent reaction product within the emulsion droplet, ensuring a reliable screening analysis. This incurs, however, an inability to control the exchange of molecules with the surrounding environment. Thus, for instance, biochemical reactions that require the addition or removal of small exogenous substrates are precluded, and analysis of binding reactions is not possible. As a consequence, evolvable phenotypes within emulsion droplets are restricted to fluorescent proteins and catalytic activities for which a fluorogenic substrate is available. Moreover, if the catalyst involved is characterized by very low catalytic efficiency, the amount of catalyst produced by a single compartmentalized cell may be insufficient to generate a readily detectable signal output. This is especially true if low sensitive readouts (e.g. absorbance) have to be used [21]. Cell amplification to colonies may be required to obtain increased levels of catalyst before the detection reaction is initiated. To date, only a few examples of cell proliferation within emulsion droplets have been reported [17, 22] and, unless chemostats are used in the microdroplets [23], the limited amount of growth medium and the accumulation of secondary metabolites in the droplets prevent high cell density cultivation. Furthermore, since enzymes are often expressed in the cells, they may not be accessible for substrates, unless those substrates are able to cross the membrane. In that case, cells must be at least partially lysed, which is difficult to achieve and control in droplet emulsions without disrupting the emulsion. Thus, control of the concentration of catalyst encountering substrate is often difficult or impossible.

Hydrogels are three-dimensional networks of hydrophilic polymers dispersed in a liquid. Due to their many advantages, hydrogels have attracted increased attention in the scientific community and have been used for many biological and biomedical applications including drug delivery, single molecule and cell analysis and tissue engineering [24–27]. Unlike water-in-oil emulsions, the porous structure of hydrogels allows the exchange of nutrients and waste to support cell growth as well as reagents to perform reactions, while preventing cells or colonies from leaking out. Hydrogels can be modified to generate structures with novel functional properties. For example, the stepwise deposition of oppositely charged polyelectrolytes via layer-by-layer (LBL) assembly on the hydrogel surface has been used to adjust hydrogel permeability rationally [28, 29].

To our knowledge, only one study so far has used hydrogel-shelled microdroplets surrounded with a polyelectrolyte coating for high-throughput enzyme screening [30]. In this study, using a microfluidic device, the authors generated emulsion droplets containing agarose and alginate which enclosed a single cell, a lysis agent and the fluorogenic substrate. Breaking the emulsion in the presence of the polycation poly(allylamine hydrochloride) (PAH) followed by subsequent deposition of poly(styrene sulfonate) (PSS) resulted in gel-shell beads (GSBs) which were successfully applied to perform directed evolution of a phosphotriesterase. Although the GSBs size selectivity enables exchange of small molecules with a molecular cutoff of ~ 2 kDa, the enzymatic reaction was conducted within emulsion droplets and relied on the compartmentalized fluorogenic substrate. The function of the polyelectrolyte shell was limited to retaining the fluorescent reaction product and to “immortalizing” emulsion droplets that could be screened by fluorescence-activated cell sorting (FACS). In our work, we extend this approach and report on the development of a novel microcompartment-based method for high-throughput screening of phenotypes that require the addition or removal of small molecule substrates. The core of the technology is the use of hollow-core polyelectrolyte-coated chitosan alginate microcapsules (HC-PCAMs) with a diameter of ~ 360 µm and a volume of ~ 25 nl. HC-PCAMs combine the advantages of hydrogel microcapsules for spatial separation and cultivation of cellular clones with the polyelectrolyte layer-by-layer technology for rational adjustment of microcapsule permeability. We show that single *E. coli* cells can be encapsulated, grown to high cell density and lysed within HC-PCAMs to generate monoclonal cell lysates that are suitable for high-throughput screening using the Complex Object Parametric Analyzer and Sorter (COPAS). We show that HC-PCAMs have a size-selective permeability that allows exchange of small molecule substrates. We therefore demonstrate the feasibility of using colonized HC-PCAMs as reaction compartments using the transpeptidation activity of the bond-forming enzyme sortase F from *P. acnes* as a model.

## Results and discussion

### Preparation of hollow-core polyelectrolyte-coated chitosan-alginate microcapsules (HC-PCAMs)

**Fig. 1:**
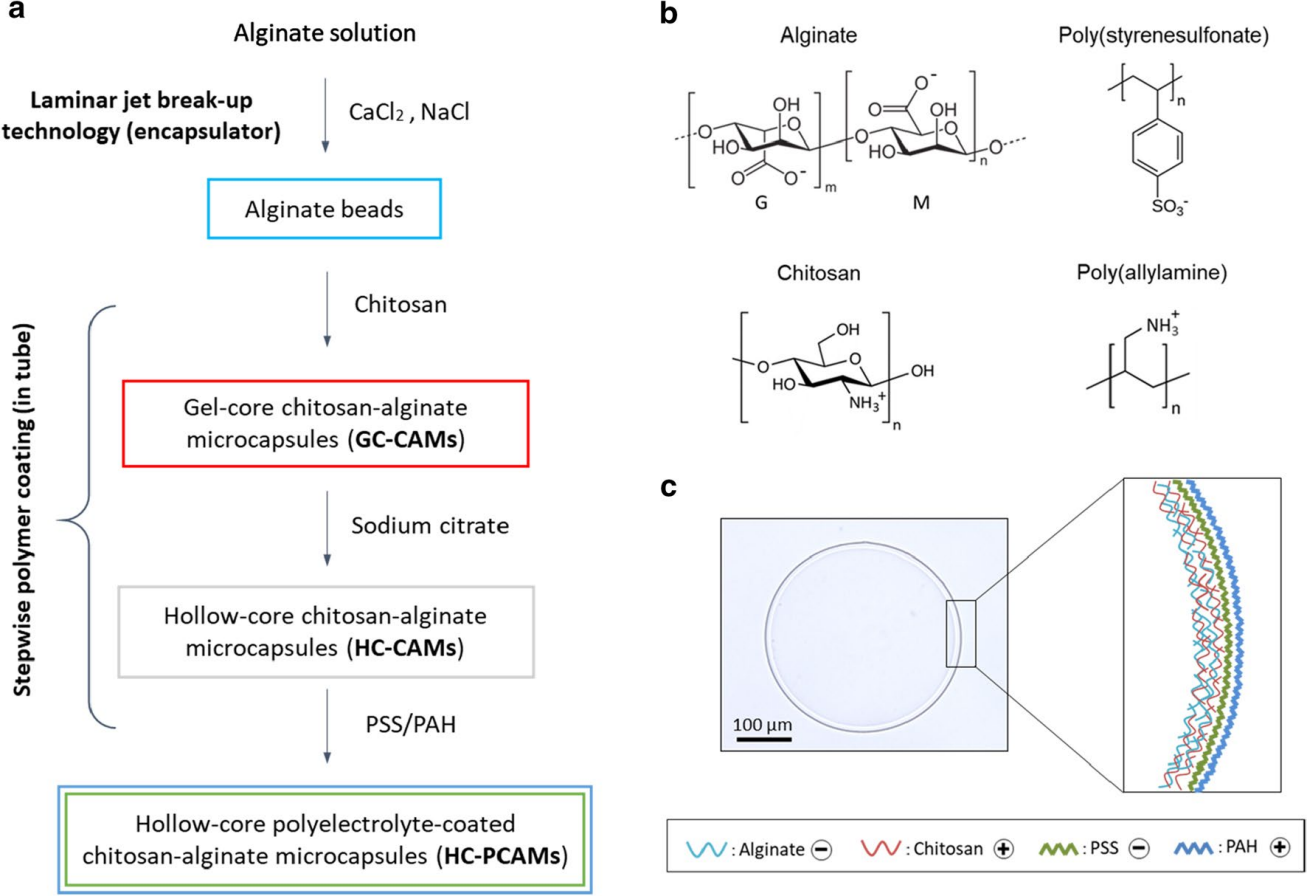
Preparation and Structure of Microcapsules developed in this Study. **a** Schematic representation of the HC-PCAMs preparation process. **b** Chemical structure of the polymers at neutral pH. G and M represent the guluronic and mannuronic acid blocks in alginate. **c** Microscopy image of a single HC-PCAM and schematic illustration of the polyelectrolyte shell composition

We prepared hollow-core polyelectrolyte-coated chitosan-alginate microcapsules (HC-PCAMs) in a four-step process (Fig. 1a). First, alginate beads were produced by laminar jet break-up technology at a rate of ~ 2500 beads/s. Next, the beads were suspended in a solution of chitosan. Adsorption of the positively charged polymer chitosan (cf. Fig. 1b) onto the negatively charged alginate leads to formation of a polyelectrolyte complex membrane at the surface of the bead of increasing thickness with longer incubation time [31, 32]. After 2 min’ incubation, the thickness of the membrane was ~ 15 µm. Subsequently, the chitosan-coated alginate beads were treated with sodium citrate resulting in dissolution of the alginate gel core while the alginate and chitosan membrane remained intact. Onto these hollow-core chitosan-alginate microcapsules (HC-CAMs) we then deposited the polyelectrolytes PSS and PAH leading to HC-PCAMs. We used bright-field microscopy and COPAS to analyze the morphology and size distribution of HC-CAMs. Spherical microcapsules with an average diameter of ~ 360 µm and average core volume of ~ 25 nl were obtained (Fig. 1c). The size distribution—calculated by COPAS as coefficient of variation—ranged between 5 and 10%, demonstrating that we achieved uniform-sized capsules with the procedure developed.

### The polyelectrolytes PSS/PAH reduce capsule permeability

**Fig. 2:**
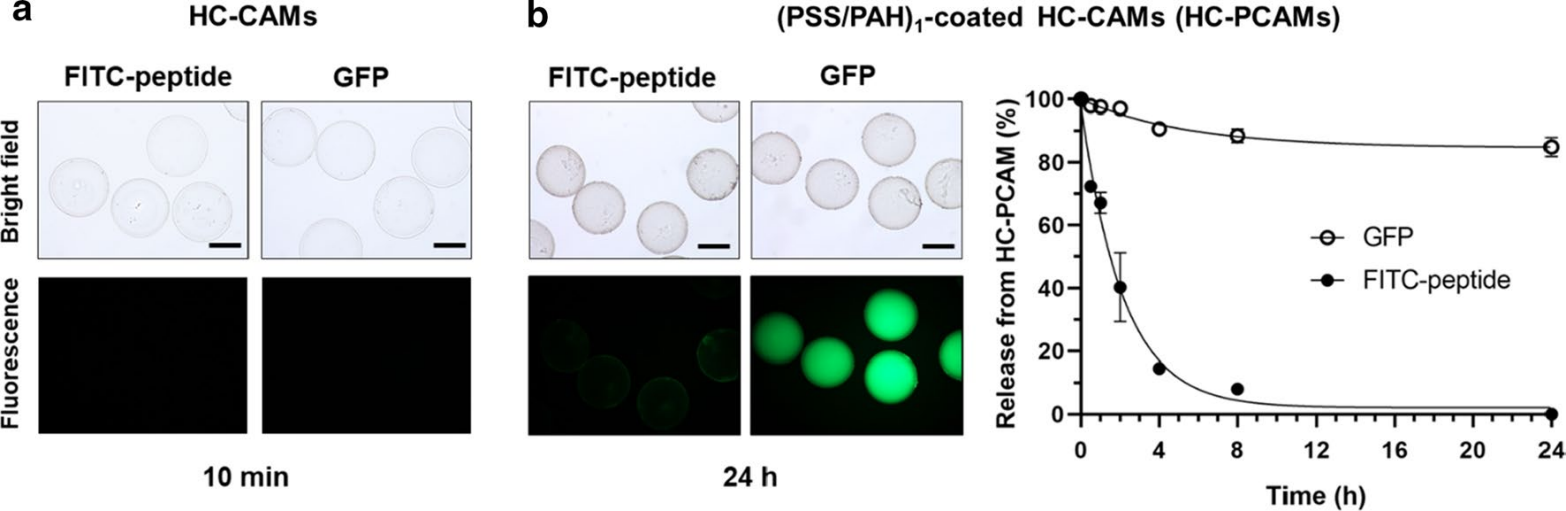
Polyelectrolyte Coating modifies Microcapsule Permeability. HC-CAMs uncoated and coated with PSS/PAH were loaded either with FITC-peptide or with GFP. The release of the two molecules from HC-CAMs and HC-PCAMs was analyzed upon incubation in a Tris buffer solution. **a** HC-CAMs loaded with either FITC-peptide or GFP exhibited no fluorescence after 10 min’ incubation when analyzed by fluorescence microscopy. The scale bar corresponds to 200 µm. **b** HC-PCAMs loaded with FITC-peptide exhibited no fluorescence after 24 h’ incubation, while those loaded with GFP exhibited a strong fluorescence signal, showing that HC-PCAMs are permeable to FITC-peptide but efficiently retain GFP (*left panel*). Kinetic release profiles of FITC-peptide (*black circles*) and GFP (*open circles*) in the Tris buffer solution from FITC-peptide and GFP-loaded HC-PCAMs respectively. Samples of buffer were taken at different time points (0, 0.5, 1, 2, 4, 8, 24 h) and fluorescence was measured using a plate reader. Data were plotted against time and fitted using the exponential decay equation (*right panel*) n = 2

In a previous study, chitosan-alginate microcapsules prepared using similar conditions showed permeability to IgG (156 kDa, hydrodynamic diameter ~ 12 nm) that suggested an average capsule pore diameter of ~ 9 nm [32]. Depending upon the subsequent application, a less permeable capsule wall may be required. Layer-by-layer coating with the polyelectrolyte pair PAH and PSS has been shown to reduce hydrogel capsule permeability for high-throughput screening applications [29, 30]. We decided to test whether this approach could also reduce permeability of the chitosan-alginate microcapsules (HC-CAMs). We assessed the permeability of the microcapsules with two fluorescent molecules, a labelled peptide (FITC-LPETGE, ~ 1 kDa) and green fluorescent protein (GFP, 30 kDa), by monitoring their diffusion out of the microcapsules by fluorescence microscopy. After 10 min incubation, HC-CAMs exhibited no fluorescence when analyzed by fluorescence microscopy, showing that they rapidly released both molecules in the incubation buffer (Fig. 2a). On the other hand, HC-PCAMs obtained by coating HC-CAMs with a single polyelectrolytes pair layer (PSS/PAH)_1_ remained permeable to the small FITC-peptide but efficiently retained GFP (Fig. 2b, *left panel*). The kinetic release profiles of FITC-peptide and GFP from HC-PCAMs showed that only ~ 10% of the GFP diffused during 24 h’ incubation while ~ 90% of FITC-peptide was already released after 4 h (Fig. 2b, *right panel*). These experiments confirmed successful deposition of PSS and PAH on the surface of HC-CAMs and demonstrated that microcapsule permeability can by controlled by deposition of PSS/PAH layers. The HC-PCAMs produced still allow small molecules (~ 1 kDa) to diffuse across the polyelectrolyte shell but large molecules (~ 30 kDa, hydrodynamic diameter ~ 5 nm) are efficiently retained inside the microcapsules.

### Microcapsules as monoclonal cell lysate compartments

Among the various applications of microcapsules, they offer the possibility to encapsulate single bacterial clones and individually study the physiology of each clone in a highly parallel fashion. We were interested in using the microcapsules for functional enzyme screening in directed evolution experiments and therefore investigated in more detail the suitability of the microcapsules for single cell encapsulation, cell proliferation and cell lysis.

**Fig. 3:**
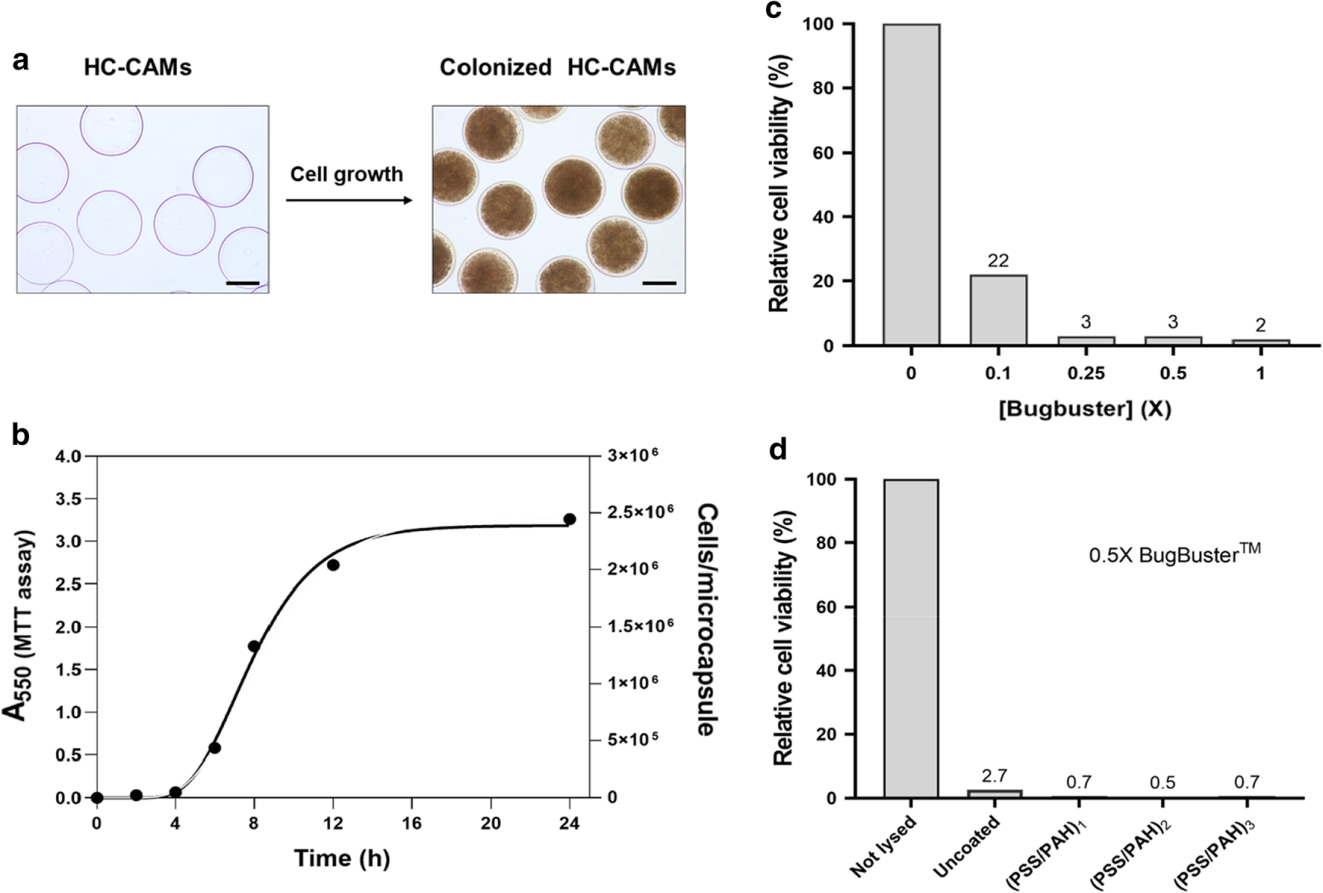
Cell cultivation and lysis within microcapsules. **a** Microscopy images of HC-CAMs before and after cell growth. The scale bar corresponds to 200 µm. **b** Growth kinetics of HC-CAMs-encapsulated *E. coli* cells as a function of time. Data were fitted using the Gompertz equation. **c** Lysis efficiency of *E. coli* cells encapsulated in HC-CAMs was determined by cell viability upon treatment with increasing BugBuster concentration. **d** BugBuster induced lysis efficiency of encapsulated *E. coli* in microcapsules with increasing number of PSS-PAH layers

For single cell encapsulation, we chose the widely used gram-negative model organism *E. coli.* Bacteria were suspended in the bulk alginate solution and hollow-core chitosan-alginate microcapsules (HC-CAMs) were prepared as described above. When cell-encapsulating HC-CAMs were prepared and transferred into the growth medium, cells proliferated to form colonies extended over the whole capsule core (Fig. 3a).

The probability of encapsulating zero, one, two or more cells in a single microcapsule is governed by the Poisson distribution [33] (Additional file 1: Figure S1a). Using a cell density of one cell per 25 nl (i.e. on average one viable bacterium per microcapsule) about one third of the microcapsules do not contain bacteria (empty capsules), one third contain a single bacterial clone (monoclonal capsules) and the remaining capsules contain two or more clones (polyclonal capsules). Because polyclonality may lead to ambiguous results during microcapsule analysis and screening, obtaining a high fraction of monoclonal microcapsules is highly desirable. This can be achieved by reducing the average number of viable bacteria per microcapsule. However, this will significantly increase the number of empty microcapsules meaning larger production volumes and longer processing times. This problem was addressed in a previous study, by enriching alginate microbeads colonized by green fluorescent *E. coli* from empty microbeads using COPAS [34]. In our work, we found that colonized hollow-core microcapsules can be efficiently enriched by gravity separation, because of their higher density compared to empty microcapsules (Additional file 1: Figure S1b).

We quantified the number of viable cells per microcapsule by comparing the metabolic activity of the encapsulated cells with that of *E. coli* cells grown in suspension via an MTT activity assay [35]. Metabolically active cells reduce the water-soluble yellow substrate MTT [3-(4,5-dimethylthiazol-2-yl)-2,5-diphenyl tetrazolium bromide] to the water-insoluble purple product formazan. After dissolution in an organic solvent, the formazan formed can be quantified spectrophotometrically and the concentration is directly proportional to the number of active cells.

After 24 h’ growth at 37 °C, a single microcapsule can host on average ~ 2.5 million cells, equivalent to a cell density of 1.3 × 10^11^ cells per ml (OD_600_ ≈ 650) (Fig. 3b). The high local cell density demonstrates that nutrients and oxygen can easily diffuse through the microcapsule shell.

Next, we investigated whether the encapsulated cells can be lysed within the microcapsules without destroying the microcapsules. We treated the microcapsules with the detergent mixture BugBuster™ (Millipore) and assessed lysis efficiency by quantifying cell viability with the MTT activity assay. Cell lysis is already efficient at a concentration of a quarter of the recommended concentration (0.25X) (Fig. 3c). Moreover, lysis is equally efficient in microcapsules coated with an increasing number of PSS-PAH layers (Fig. 3d), confirming the high permeability of the capsules for low molecular weight compounds. Microscopic inspection of microcapsules after cell lysis revealed that neither cell growth nor detergent treatment affected the integrity of the microcapsules.

### Sortase-mediated conjugation within HC-PCAMs

Sortases are a family of bacterial enzymes that are responsible for attachment of secreted proteins to the cell wall via a transpeptidation reaction. These cysteine transpeptidases first recognize and cleave a five amino acid long sorting motif on the target proteins (e.g. LPXTG, where X is variable) to generate a thioester-linked acyl enzyme intermediate. They then catalyze the formation of a new peptide bond between the cleaved sorting motif (the acyl donor) and the free amino group of a cell wall component (the acyl acceptor) (Additional file 1: Figure S2a). In the last decade, the transpeptidation reaction catalyzed by sortases has attracted increasing attention in the scientific community and has been exploited for the site-specific conjugation of a variety of acyl donor and acceptor substrates. This process, also referred to as sortagging [36] has been used for a wide range of biotechnological and biomedical applications including protein functionalization [37–39], protein–protein ligation [40], protein immobilization [41, 42] and cyclization [43, 44]. However, the widespread use of sortases for sortagging is being hampered by the intrinsic low activity of this family of enzymes. The use of sortases for sortagging gained significance after the development of an enhanced version of the most active and best characterized sortase A from *S. aureus* (SaSrtA) by directed evolution [45]. Due to the very low activity, sortases from classes other than class A are not currently used for sortagging applications and, apart from SaSrtA, in vitro evolution of other sortases has not yet been reported.

In a recent work, we reported that sortase F from *Propionibacterium acnes* (PaSrtF) catalyzes the transpeptidation of peptides bearing the **LPQTG**E motif (the SrtF recognition sequence is highlighted in bold) to N-terminally oligo-glycine modified molecules acting as acyl acceptors in the reaction [46]. To demonstrate the feasibility of using HC-PCAMs as reaction compartments for high-throughput screening procedures, we used PaSrtF as a model and assessed whether the SrtF-catalyzed transpeptidation reaction could be assayed efficiently and reliably within microcapsules.

For analysis of individual microcapsules a fluorescent readout is required. We therefore used the SrtF recognition peptide substrate (LPQTGE) labelled at the N-terminus with fluorescein isothiocyanate (FITC), and adapted a previously reported product capture strategy [47] that relies on a sortase enzyme harboring a glycine at its N-terminus. In the course of the sortase reaction the fluorescent peptide is covalently linked to the SrtF which serves as enzyme and whose N-terminus is the acceptor substrate (Additional file 1: Figure S2b).

**Fig. 4:**
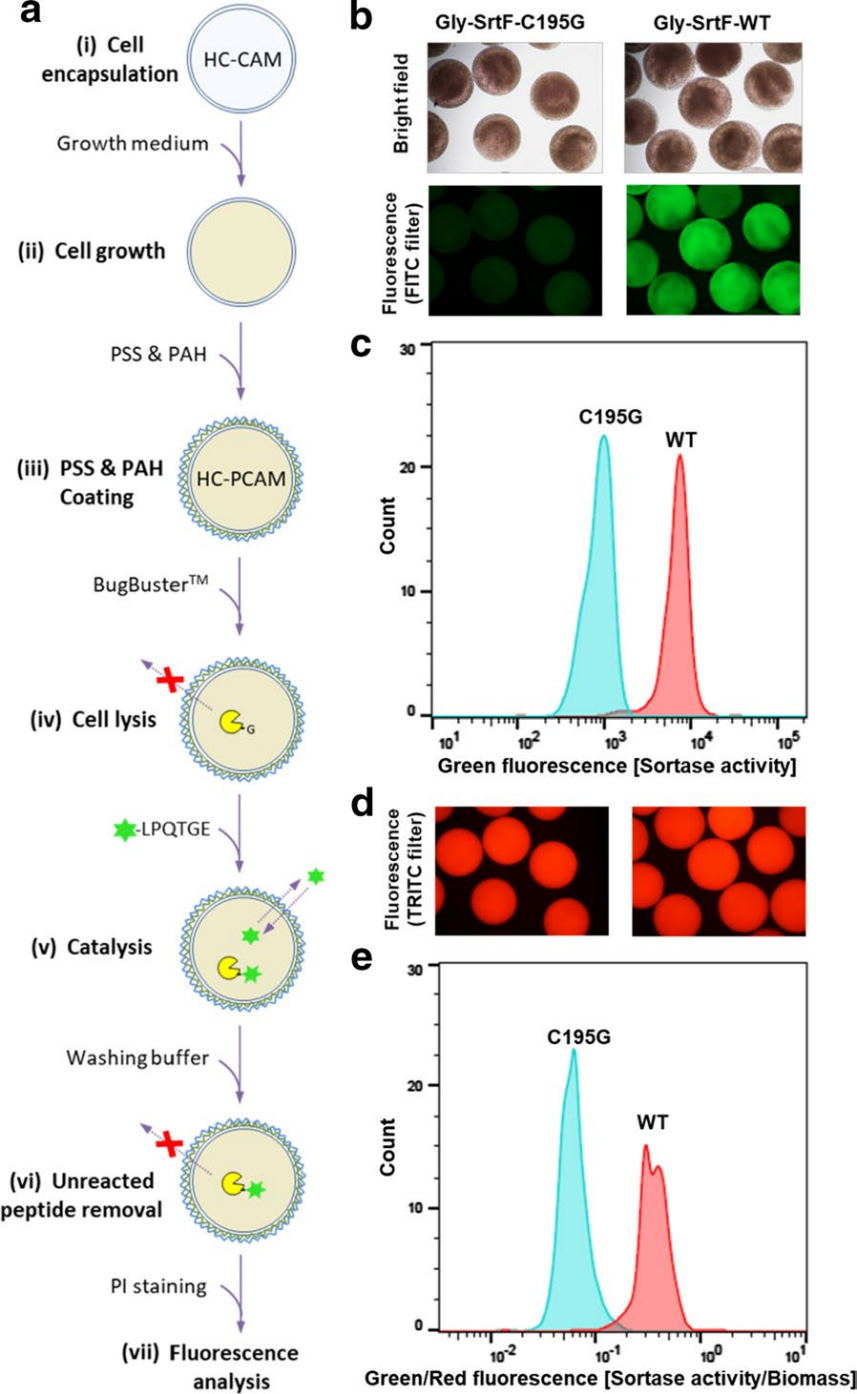
SrtF transpeptidation activity can be assayed within HC-PCAMs. **a** Workflow of the HC-PCAMs-based method for the analysis of SrtF activity. (i) *E. coli* cells, harboring the plasmid encoding either the wild type Gly-SrtF (WT) or the inactive mutant Gly-SrtF-C195G (negative control), are encapsulated within HC-CAMs. (ii) Microcapsules are transferred into the growth medium to allow cells to grow to colonies that express the respective enzymes. (iii) Colonized HC-CAMs are coated with PSS and PAH to reduce the permeability and generate colonized HC-PCAMs. (iv) Upon cell lysis, the expressed N-terminally glycine-modified sortase is released from cells but retained within HC-PCAMs. (v) Microcapsules are then incubated with the fluorescent peptide. Due to the low molecular weight, the fluorescent peptide can permeate through the polyelectrolyte shell and initiate the sortase-mediated transpeptidation reaction. (vi) Upon washing, the unreacted fluorescent peptide is removed and the reaction stopped. The polyelectrolyte coating retains the fluorescent transpeptidation product within HC-PCAMs, allowing detection of sortase activity via fluorescence analysis. (vii) Microcapsules are incubated with the fluorescent DNA intercalating agent propidium iodide (PI) and analyzed for fluorescence by fluorescence microscopy and COPAS. **b** Bright field (*upper panel*) and green fluorescence (*lower panel*) pictures of reacted HC-PCAMs showing successful conjugation and capture of the fluorescent peptide within HC-PCAMs expressing the wild type Gly-SrtF but not within HC-CAMs expressing the inactive enzyme. **c** Histogram overlays of HC-PCAMs analyzed with COPAS showing green fluorescence distribution (sortase activity) of Gly-SrtF wild type (red) and Gly-SrtF-195G (cyan) populations. **d** Red fluorescence pictures of HC-PCAMs after the addition of propidium iodide. **e** Histogram overlays of COPAS analyzed HC-PCAMs showing the distribution of the two HC-PCAMs populations after normalization of the green fluorescence signal (sortase activity) for the red fluorescence signal (lysed biomass) obtained with propidium iodide

In fact, in crude cell extracts containing recombinant SrtF, modified by the addition of a single glycine residue at the N-terminus (Gly-SrtF), SrtF is efficiently labelled in the presence of the fluorescent recognition peptide (data not shown). We investigated whether the same reaction could take place inside microcapsules containing lysates from recombinant *E. coli* cells. The permeability of the HC-PCAMs would allow the fluorescent recognition peptide to diffuse inside and take part in the sortase-mediated conjugation reaction while preventing Gly-SrtF and its transpeptidation product (~ 22 kDa) from diffusing out. To this end, *E. coli* cells, harboring the plasmid encoding either Gly-SrtF wild type or the inactive variant Gly-SrtF-C195G (used as negative control), were encapsulated within HC-CAMs and grown to generate microcapsules in which the respective enzyme is expressed. Following coating with the polyelectrolyte PSS and PAH, microcapsules were treated with BugBuster to achieve cell lysis. Subsequently, the microcapsules were incubated in the reaction buffer supplemented with the fluorescent substrate to initiate the sortase-mediated conjugation reaction. After the reaction, microcapsules were washed to remove unreacted peptide substrate and analyzed by fluorescence microscopy and COPAS. The workflow of the assay is schematized in Fig. 4a. In comparison to microcapsules expressing the inactive Gly-SrtF-C195G, microcapsules expressing Gly-SrtF wild type exhibited a significant increase of the green fluorescence signal when analyzed by fluorescence microscopy (Fig. 4b) and quantitative COPAS analysis (Fig. 4c), indicating a robust and consistent conjugation reaction within the microcapsules. To normalize the activity signal (green fluorescence of the captured and retained peptide) against the lysed biomass, we stained the microcapsules with the fluorescent DNA intercalating agent propidium iodide (PI) before COPAS analysis, which could be detected as red fluorescence signal. When the COPAS analysis was performed with the normalized signal (green/red fluorescence) the population of microcapsules containing the Gly-SrtF wild type was clearly separated from the population containing the inactive mutant (Fig. 4d, e).

**Fig. 5:**
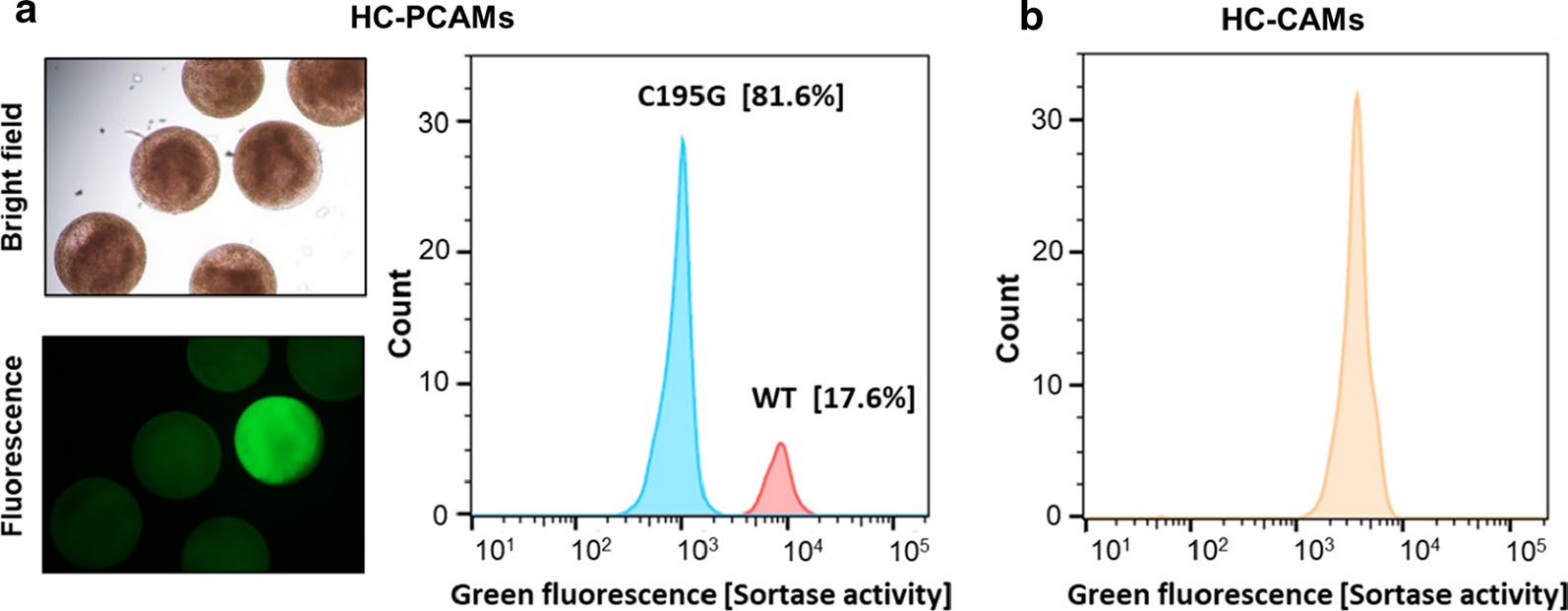
HC-PCAMs behave as individual reaction compartments that can be distinguished based on enzymatic activity. **a** Microscopic (*left side*) and COPAS analysis (*right side*) of mixtures of HC-PCAMs (1:5 ratio of microcapsules with cells expressing Gly-SrtF wild type:Gly-SrtF-C195G) after 21 h’ reaction with 50 µM FITC-LPQTGE. Consistent with the mixing ratio, the analysis yielded two well-separated microcapsule populations. Microcapsules expressing the wild type enzyme were clearly distinguishable from those expressing the inactive mutant. **b** Unlike HC-PCAMs, non-coated microcapsules exchanged cell lysates and reaction products and yield a single population with an intermediate green fluorescence intensity

To verify that the method can be used to sort microcapsules based on different enzymatic activities, we mixed HC-PCAMs in a 1:5 ratio of Gly-SrtF wild type:Gly-SrtF-C195G (with Gly-SrtF-C195G being the inactive variant of SrtF). The microcapsules in the mixture were coated with PSS and PAH and the sortase-mediated conjugation reaction was performed. As shown in Fig. 5a, the fluorescence microscopy and COPAS analysis of the sample with mixed microcapsules revealed two distinct populations. One population (~ 18%) exhibited a significantly increased level of fluorescence (about one order of magnitude) while the second (~ 82%) showed only background fluorescence. This result is consistent with the mixing ratio and shows that microcapsules expressing the wild type enzyme can be clearly distinguished from those expressing the inactive mutant according to the fluorescence intensity. Furthermore, this experiment demonstrates that the method can be used to enrich or sort specific clones from a library. In support of this result, we isolated members of the active population, recovered the plasmid DNA which was then transformed into *E. coli* cells by electroporation (“HC-PCAMs efficiently retain the plasmid DNA but allow its recovery” section) and after plasmid isolation their identity was confirmed by DNA sequencing.

On the other hand, when the same experiment was performed with a mixture of non-coated microcapsules containing either bacteria expressing wild type Gly-SrtF or Gly-SrtF-C195G, the COPAS analysis yielded a single peak in which the wild type and the inactive populations cannot be distinguished (Fig. 5b). This result clearly shows that unlike non-coated microcapsules, HC-PCAMs behave as individual reaction compartments that efficiently retain the lysates and the reaction products, preventing their exchange between different microcapsules.

### HC-PCAMs efficiently retain the plasmid DNA but allow its recovery

The successful application of artificial microcompartments for directed evolution of proteins requires that the phenotype exhibited by a protein remains linked to the genotype in order to retrieve the genetic information. This implies that the DNA molecule encoding the protein is retained inside the compartment throughout the duration of the screening procedure and is eventually recovered for protein variant identification and, if necessary, for additional rounds of evolution.

**Fig. 6:**
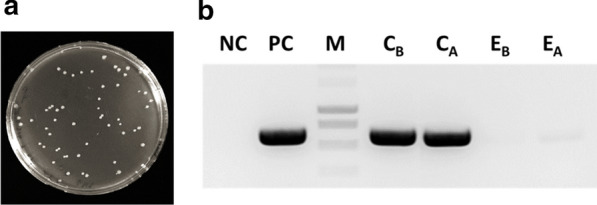
Genetic information can be recovered from a single HC-PCAM. **a** LB agar plate showing transformants after electroporation of isolate from a single microcapsule into *E. coli* cells. **b** PCR amplification of the SrtF gene from colonized (C) and empty (E) microcapsules taken before (C_B_ and E_B_) and after (C_A_ and E_A_) the sortase-mediated conjugation, showing that the plasmid is not significantly exchanged between microcapsules. NC: Negative control (No template), *PC* positive control, *M* DNA ladder, *C* colonized microcapsule

To recover the plasmids, HC-PCAMs were dissolved by treatment with a solution of sodium hydroxide. The treatment disassembles the polyelectrolyte complex shell and leads to dissolution of the microcapsule, liberating the enclosed DNA plasmid. The obtained solution containing the plasmid was then either transformed into bacteria for in vivo amplification (Fig. 6a) or used for PCR to amplify the gene of interest (Fig. 6b). Both methods can be used to retrieve genetic information, but direct transformation of the plasmid into cells is considerably faster than PCR amplification and subsequent cloning. In addition, direct transformation does not pose the risk of introducing new mutations as can occur using PCR.

To verify that the plasmid is efficiently retained inside microcapsules and is not exchanged between them during the assay, we assessed whether the wild type SrtF gene enclosed within colonized HC-PCAMs could be amplified by PCR from the empty microcapsules also present in the sortase-mediated conjugation reaction. If the plasmid is exchanged between microcapsules, we would expect PCR amplification of the SrtF gene from both colonized and empty microcapsules. In fact, as shown in figure Fig. 6b, PCR products were obtained from colonized microcapsules but no significant PCR product was obtained from the reaction with extracts from empty microcapsules. This result demonstrates that HC-PCAMs efficiently retain the plasmid and do not exchange it during the whole assay duration. Most importantly, the plasmid DNA can be retrieved from the microcapsules for further processing.

## Conclusions

We have developed an innovative high-throughput screening method that relies on the use of a novel type of polymer hydrogel microcapsule. Characterized by size-selective permeability, these microcapsules serve as miniature cell culture and reaction compartments suitable for high-throughput screening of bacterial cell libraries whose assay format requires small molecule substrates to enter or leave the capsule. We have demonstrated that such microcapsules act as individual evolutionary units that link the transpeptidation activity of sortase enzymes with a fluorescence readout. The capsule permeability permits a fluorescently labelled peptide substrate to diffuse inside the capsule and take part in the sortase-mediated reaction; however, it prevents the sortase enzyme and the fluorescent reaction products from leaking out. Using a COPAS particle analyzer, we showed that clones can be analyzed with high-throughput (~ 10^5^/h) according to sortase activity, making the assay a powerful tool for screening sortase libraries in directed evolution experiments.

The method is compatible with bacterial cell lysate; however, it could be applied to different cell types (e.g. yeast, mammalian cells) and many kinds of protein expression systems including in vitro transcription/translation, cell display or cell secretion. Notably, the possibility to grow cells to high density within hollow-core microcapsules allows maximizing the amount of protein that can be assayed in a single microcompartment. Apart from fluorescence-based systems, this would enable downward extension of the dynamic range of screening methods that rely on less sensitive readouts (e.g. absorbance) thereby making enzymes accessible for directed evolution even if they show very low activity or detection exhibits low sensitivity. Furthermore, cell colonies immobilized in selective permeable microcapsules provide a new and efficient means for preparing biocatalysts that can be easily recovered and reused or used as biosensor agents.

The method extends evolvable phenotypes in artificial microcompartments to catalytic activities for which fluorogenic substrates cannot be made but which require the exogenous supply of small molecule substrates. Nevertheless, it is worth noting that in the presented format, the method might not be capable of screening enzymatic reactions involving molecules that can diffuse out from microcapsules. To prevent the leakage through the polyelectrolyte shell, reaction products have to be immobilized on a larger molecules or substrates yielding insoluble products have to be used.

Using different polyelectrolytes and different coating conditions, the layer-by-layer technology could be used to adjust the capsule permeability according to user needs to rationally control the diffusion of molecules inside and from microcapsules. This, in addition to catalysts, would enable the application of the method for directed evolution of binding affinities.

The method is robust, inexpensive and easy to scale-up. During each step of the procedure, microcapsules are processed in parallel, which facilitates manipulation without the need for automated systems. Based on our experience the procedure would allow processing and analysis of up to 10^6^ clones in 48–72 h. The throughput of the method could be further increased by reducing microcapsule size, allowing isolation of the microcapsules with fluorescence activated cell sorting (FACS) instead of COPAS. For instance, microfluidics systems have been developed that allow the generation of uniform alginate microbeads as small as 10 µm in diameter which encapsulate cells with high viability [48, 49]. By adjusting coating conditions, HC-PCAMs might be prepared from microfluidics-based alginate microbeads, allowing screening and selection by FACS at rates of up to 10^7^ per hour.

## Methods

### Preparation of alginate beads

Alginate beads were prepared using the semi-automated encapsulator B-395 Pro (Encapsulator Biotech/Buchi). A solution of 3% low viscosity sodium alginate (no. A112, Sigma-Aldrich) was prepared in 25 mM Tris/HCl pH 7.8. The solution was centrifuged for 10 min at 5,000*g* and filter-sterilized with a 0.22 µm membrane filter (steriflip, Millipore). The alginate solution was transferred into a 20 ml syringe and extruded through a 150-µm diameter nozzle into a gently stirred beaker filled with 20 volumes of 25 mM Tris/HCl pH 7.8, 50 mM CaCl_2_, 200 mM NaCl, 0.1% Tween 20. Uniform and monodispersed beads were produced at a rate of 2480 Hz (Amplitude = 5) with a flow rate of 3.12 ml/min and an electrode tension of 1080 V. Alginate beads were allowed to harden for 15 min, collected at the bottom of a 50 ml tube by sedimentation and washed/re-suspended twice with 20 mM Tris/HCl pH 7.8, 100 mM NaCl, 10 mM CaCl_2_, 0.05% Tween 20 (40 ml).

### Preparation of hollow-core chitosan alginate microcapsules

A chitosan solution (0.3% w/v) was prepared by adding low molecular weight chitosan (no. 448869, Sigma-Aldrich) to 25 mM sodium acetate pH 5.0, 200 mM CaCl_2_. After 30 min of stirring, the pH was adjusted to 5.0 with glacial acetic acid and following centrifugation at 15,000*g* for 20 min the supernatant was recovered and autoclaved. *Gel*-*core chitosan*- *alginate microcapsules* (GC-CAMs) were obtained by adding alginate beads to two volumes of gently stirred chitosan solution and incubation for two minutes.

GC-CAMs were collected in a 50 ml tube and washed/re-suspended twice with 10 volumes of 100 mM NaCl, 10 mM CaCl_2_, 0.05% Tween 20 to remove excess of chitosan. Next, GC-CAMs were re-suspended with five volumes of the latter solution supplemented with 20 mM sodium citrate for 1 min to dissolve the alginate gel-core and generate *hollow*-*core chitosan*-*alginate microcapsules* (HC-CAMs). HC-CAMs were washed once with 10 volumes of 100 mM NaCl, 10 mM CaCl_2_, 0.05% Tween 20 to remove the sodium citrate.

### Polyelectrolyte coating onto hollow-core chitosan alginate microcapsules

HC-CAMs were used as a template for stepwise deposition of the polyelectrolyte pair poly(styrene sulfonate) (PSS, no. 243051, Sigma-Aldrich) and poly(allylamine hydrochloride) (PAH, no. 283223 Sigma-Aldrich). HC-CAMs (~ 100 μl settled volume, ~ 3000 microcapsules) were transferred into 2 ml centrifuge tubes and incubated with five volumes (500 μl) of 2 mg/ml PSS in 0.5 M NaCl, 0.5 M CaCl_2_ for 10 min at 25 °C and 850 rpm shaking. Before proceeding with the deposition of PAH or of subsequent polyelectrolyte layers the excess polyelectrolyte was removed by three washing/re-suspension steps with ~ 20 volumes of 100 mM NaCl, 10 mM CaCl2, 0.05% Tween 20 (2 ml final volume) for 1 min and 850 rpm shaking. PSS-coated HC-CAMs were then coated with PAH by incubation into five volumes (500 μl) of 2 mg/ml PAH in 0.5 M NaCl) for 10 min at 25 °C and 850 rpm shaking. Multilayered microcapsules were prepared by depositing additional layers of PSS and PAH with the same procedure described above. At the end of the coating procedure, the resulting *hollow*-*core polyelectrolyte*-*coated chitosan*-*alginate microcapsules* (HC-PCAMs) with one or multiple PSS-PAH layers were likewise washed with 100 mM NaCl, 10 mM CaCl2, 0.05% Tween 20 to remove excess PAH.

### Analysis of microcapsule permeability

Samples of HC-CAMs (~ 100 μl settled volume) were incubated with 500 μl of 100 mM NaCl, 10 mM CaCl2 0.05% Tween 20 (500 μl) either containing 2 µM of a fluorescein isothiocyanate (FITC)-labelled peptide (FITC-LPETGE, Genscript) or Green Fluorescent Protein (GFP, Uniprot: P42212) with shaking at 600 rpm for 15 min. FITC-peptide and GFP-loaded HC-CAMs were coated with a layer of PSS and a layer of PAH following the procedure described above. In this case, both the two polyelectrolytes and washing solutions were supplemented with either 2 µM of FITC-peptide or GFP for the coating of FITC-peptide or GFP-loaded microcapsules respectively. The resulting FITC-peptide and GFP-loaded HC-PCAMs were re-suspended in ~ 20 volumes of 25 mM Tris/HCl pH 8.2, 150 mM NaCl, 10 mM CaCl_2_ (2 ml final volume) and the release of the two fluorescent molecules from HC-PCAMs into the incubation buffer was monitored by fluorescence microscopy. Kinetic release profiles of FITC-peptide and GFP from FITC-peptide and GFP-loaded HC-PCAMs, respectively, were obtained by measuring the fluorescence of incubation buffer samples withdrawn over a period of 24 h using a fluorescence microplate reader (Infinite M200, TECAN; Ex: 490 nm, Em: 520 nm). As a control, the release of the two fluorescent molecules was analyzed from microcapsules without the PSS/PAH shell.

### Cell encapsulation, growth and lysis within microcapsules

For cell encapsulation, *E. coli* BL21 (DE3) cells harboring the plasmid of interest from cultures in the exponential growth phase were diluted to an OD_600_ of 0.1 with 50 mM Tris/HCl pH 7.6, 100 mM NaCl. An aliquot of this cell suspension was added to the alginate solution to achieve an average of 1 or more encapsulated cells per microcapsule. After mixing for 1 min, the alginate solution containing cells was transferred into a 20 ml syringe and GC-CAMs were prepared as described above. GC-CAM-entrapping cells were transferred into a conical flask filled with terrific broth medium (25 ml) supplemented with 20 mM sodium citrate to dissolve the alginate gel core and cells were grown for 20 h at 28 °C and 150 rpm shaking. The analysis of encapsulated cells which eventually developed into colonies showed that more than 80% of encapsulated cells survive the encapsulation procedure. Protein expression was induced by the addition of 0.2 mM IPTG for 20 h at 16 °C. After cell growth and protein expression, microcapsules were washed/re-suspended three times with 10 volumes of 100 mM NaCl, 10 mM CaCl_2_, 0.05% Tween 20, before proceeding with the deposition of layers of PSS and PAH.

Cell growth kinetics within microcapsules were determined by evaluating the cell viability of encapsulated cells grown at 37 °C by using an MTT reduction assay [35]. Microcapsules were taken at different time points and washed with 100 mM NaCl, 10 mM CaCl_2_, 0.05% Tween 20 to remove traces of growth medium and possible *E. coli* cells grown in suspension. Samples of cell-encapsulating microcapsules (~ 10 µl settled volume, ~ 300 microcapsules) were transferred into 1.5-ml tubes and the residual liquid was pipetted off. Microcapsules were suspended with pre-warmed LB medium (200 µl) and the MTT reduction reaction was initiated by the addition of MTT solution (20 μl of 5.0 mg/ml MTT in water) pre-warmed to 37 °C. Tubes were incubated at 37 °C for 15 min and 1000 rpm shaking with the tube cap open. The liquid was pipetted off and the purple formazan crystals formed inside microcapsules were dissolved with DMSO (1 ml) by vortexing for 1 min. After 10 min, the absorbance was recorded at 550 nm using a spectrophotometer. The number of viable cells per microcapsule at each time point was estimated from a standard curve obtained by plotting the MTT assay activity of *E. coli* cells grown in suspension.

Lysis of *E. coli* cells within microcapsules was achieved using the detergent mixture BugBuster™ (10 X, no. 70921, Merck Millipore). Cell lysis efficiency was assessed by evaluating the viability of encapsulated cells after the treatment with different BugBuster™ concentrations with the MTT reduction assay. Samples of cell-encapsulating microcapsules (~ 100 μl settled volume) were transferred into 2-ml tubes and incubated with 25 mM Tris/HCl pH 8.2, 150 mM NaCl, 10 mM CaCl_2_ supplemented with BugBuster™ (500 μl final volume) for 30 min at 25 °C and 850 rpm shaking. Following three washing steps with the same Tris–HCl buffer (2 ml) to remove excess detergent, microcapsules were transferred to 1.5-ml centrifuge tubes and the MTT assay was performed as described above.

### Construction of Gly-SrtF-Pa and Gly-SrtF-Pa-C195G

For recombinant expression of sortase F with a glycine at the N-terminus, the core region of sortase F from *P.* *acnes* KPA171202 (e.g. GenBank: AAT82533.1, Uniprot: Q6A9N3, from threonine 35 to alanine 217) was custom-synthesized by GenScript and cloned into a pUC57 vector. The gene was transferred to the expression vector backbone p7X yielding p7X-SrtF-Pa-strep [50]. Site-directed mutagenesis PCR was then performed with Phusion HF DNA Polymerase (NEB) using the synthetic oligonucleotides *N*-*term Gly.for* (5′-GGAGATATACATATGGGCAGTACCACCACGTCAAGCACG-3′) and *N*-*term Gly.rev* (5′-CGTGCTTGACGTGGTGGTACTGCCCATATGTATATCTCC-3′) (Microsynth). Following treatment with DpnI, *E. coli* XL1-Blue cells were transformed with the PCR reaction mixture to yield the expression vector p7X-Gly-SrtF-Pa-strep that encodes recombinant Gly-SrtF with a C-terminal twin-strep-tag under the strong T7 RNA polymerase promoter. Similarly, to obtain the inactive variant Gly-SrtF-C195G, site-directed mutagenesis PCR was performed over p7X-Gly-SrtF-Pa-strep with the synthetic oligonucleotides *SrtF*-*Pa*-*C195G.for* (5′-AGCTATTGTGATTGGCTGGGATTACGTGAAG-3′) and *SrtF*-*Pa*-*C195G.rev* (5′-CTTCACGTAATCCCAGCCAATCACAATAGCT-3′) to yield p7X-Gly-SrtF-C195G-strep. The gene sequences in the expression vectors were verified by DNA sequencing.

### Sortase-mediated conjugation within HC-PCAMs

HC-CAMs encapsulating cells harboring either the expression vector p7X-Gly-SrtF-Pa-strep or p7X-Gly-SrtF-Pa-C195G were prepared as described above. Samples of colonized microcapsules (~ 100 μl settled volume) were transferred into 2-ml tubes and incubated with 50 mM Tris/HCl pH 7.6, 500 mM NaCl, 10 mM CaCl_2_, 0.5X BugBuster™ (500 μl final volume) for 30 min at 25 °C and 850 rpm shaking. Following three washing steps with the Tris–HCl buffer (2 ml) to remove excess detergent, microcapsules were incubated with 50 µM FITC-LPQTGE in 50 mM Tris–HCl pH 7.6, 500 mM NaCl, 10 mM CaCl_2_ (500 μl) for 21 h at 25 °C. After the reaction, microcapsules were washed three times with 50 mM Glycine pH 9.5, 500 mM NaCl, 10 mM CaCl_2_, 0.1% SB3-14 (2 ml) for 5 min and 850 rpm shaking to remove unreacted fluorescent peptide and twice with 10 mM Tris/HCl pH 8.5, 10 mM CaCl_2_ (2 ml) for 1 min and 850 rpm shaking to remove excess detergent. Following incubation with 10 mM Tris/HCl pH 8.5, 10 mM CaCl_2_, 0.1 mg/ml propidium iodide (1 ml) for 5 min and 850 rpm shaking, microcapsules were washed twice with 10 mM Tris/HCl pH 8.5, 10 mM CaCl_2_ (2 ml) to remove excess propidium iodide and analyzed by fluorescence microscopy and COPAS.

### Fluorescence microscopy and COPAS analysis

Fluorescence microscopy was performed with an Olympus BX41 microscope. Non-filtered white light (bright field) was used to illuminate the microcapsule structure. Two filter sets that allowed for blue excitation and green emission (excitation filter at 480/20; long-pass emission filter at 510 nm) or green excitation and red emission (excitation filter at 535/30; long-pass emission filter at 580 nm) were used to analyze FITC and propidium iodide-based fluorescence, respectively. Pictures were taken using lenses with 10 X and 20 X magnification. Exposure times were 1/100 s for FITC-based fluorescence and from 1/2000s for propidium iodide-based fluorescence. Microcapsules were spectrophotometrically imaged with a COPAS Plus particle analyzer and sorter (1000 µm flow-cell) controlled with the Flow pilot software (Union Biometrica). Fluorescence signals were recorded at 510 nm, appropriate for the emission maximum of FITC and at 615 nm appropriate for propidium iodide [ex: 488 nm, photon multiplier settings (PMT green): 450–650 V (PMT red): 450 V; gain factor: 1.0. The COPAS-device was operated at frequencies between 30 and 40 Hz; At least 600 microcapsules were analyzed. Data analysis was performed using the FlowJo software.

### Genotype recovery

To recover the plasmid DNA, individual HC-PCAMs were collected with 1 μl of the buffer in which they were suspended (10 mM Tris/HCl pH 8.5, 10 mM CaCl_2_) by using a pipette. Each microcapsule was transferred into a 1.5-ml tube and mixed with NaOH (5 μl, 50 mM) for disassembly of the polyelectrolyte shell and microcapsule dissolution. Following 1 min vortexing and 5 min shaking at 1000 rpm, acetic acid (5 μl of 50 mM) was added to neutralize the pH and the sample vortexed again for 1 min. For plasmid transformation by electroporation, an aliquot of the sample containing the plasmid (4 μl) was mixed with 40 μl of electrocompetent BL21 (DE3) cells (CMC0016, Sigma-Aldrich) previously diluted 1 to 4 with sterile and ice cold glycerol (10%). Following incubation on ice for 1 min, cells were electroporated at 2.5 kV, 200 Ω and 25 μF using a 2 mm electroporation cuvette in a Gene Pulser Xcell unit (Biorad). After the pulse, recovery medium (960 μl, no. CMR0001, Sigma-Aldrich) was added to the cells in the cuvette and the cell suspension was transferred into a 15-ml tube. After incubation for 1 h at 37 °C and 200 rpm for cell recovery, the cell suspension was centrifuged for 3 min at 3,000*g*. Cells were spread on LB agar plate and allowed to grow overnight at 37 °C.

For the amplification of the SrtF gene by PCR, an aliquot of the isolated sample (1 μl) from either colonized or empty microcapslues was mixed with synthetic oligonucleotide primers FX_SrtE-StrepTag.for (0.5 μM; 5′-ATATATGCTCTTCTAGTACCACCACGTCAAGCACGAG-3′) and FX_SrtE-StrepTag.rev (0.5 μM; 5′-TATATAGCTCTTCATGCTTATTATTTTTCAAACTGCGGATGGG-3′) (Microsynth), Phusion HF DNA Polymerase (3 units; NEB), Phusion HF Buffer (1X, NEB) and dNTPs (200 μM). The reactions were thermocycled using the following protocol: denaturation: 95 °C for 5 min; 14 cycles at 95 °C for denaturation (30 s), 61 °C for annealing (30 s) (decrease 0.5 °C per cycle) and elongation at 72 °C (1 min); 14 additional cycles were performed at 95 °C for denaturation (30 s), 53 °C for annealing (30 s) and elongation at 72 °C (1 min). PCR reactions were analyzed on a 1% (w/v) agarose gel.

## Supplementary information

**Additional file 1: Figure S1.** Poisson Statistics and enrichment of colonized microcapsules. **a** Shown is the probability that a colonized microcapsule is monoclonal according to the Poisson distribution. During cell encapsulation, the lower the average number of viable cells per capsule (λ), the higher the probability that a colonized microcapsule is monoclonal (red). A low average number of cells per capsule leads to a lower fraction of monoclonal microcapsules (blue) and, in turn, to a higher fraction of uncolonized microcapsules. **b** Scheme of the gravity-driven separation procedure for the enrichment of colonized microcapsules from a mixture of colonized and uncolonized microcapsules. **Figure S2.** Sortase enzymes catalyze a transpeptidation reaction. **a** Scheme of the transpeptidation reaction catalyzed by sortase enzymes. Sortases recognize the pentapeptide sorting motif (e.g. LPXTG here) on the target protein (acyl donor substrate); The cysteine in the active site of the enzyme performs a nucleophilic attack on the carbonyl carbon of threonine residue, breaking the peptide bond between the threonine and the glycine and forming an intermediate in which the enzyme and the target protein are linked together via a thioacyl linkage. The subsequent nucleophilic attack of the thioacyl linkage by the free amino group of an oligoglycine stretch of a cell wall component (acyl acceptor substrate) resolves the intermediate and results to the covalent attachment of the target protein to the bacterial cell wall. **b** Scheme of the intramolecular transpeptidation reaction catalyzed by an N-terminally-modified sortase enzyme. The glycine residue at the N-terminus acts as the acyl acceptor substrate that solves the intermediate in an intramolecular fashion. This enables the sortase enzyme to covalently capture the acyl donor substrate (e.g. a FITC-labelled pentapeptide).

## Data Availability

Please contact corresponding author for data requests.

## Abbreviations

HC-CAMs: Hollow-core chitosan-alginate microcapsules
HC-PCAMs: Hollow-core polyelectrolyte-coated chitosan-alginate microcapsules
LBL: Layer-by-layer
SrtF: Sortase F
*P. acnes*: *Propionibacterium acnes*
IVC: In vitro compartmentalization
COPAS: Complex Object Parametric Analyzer and Sorter
PSS: Poly(styrene sulfonate)
PAH: Poly(allylamine hydrochloride)
GSBs: Gel-shell beads
FITC: Fluorescein isothiocyanate
GFP: Green fluorescent protein
MTT: 3-(4,5-Dimethylthiazol-2-yl)-2,5-diphenyl tetrazolium bromide
Gly-SrtF: N-terminally glycine-modified sortase F
PI: Propidium iodide
FACS: Fluorescence activated cell sorting
