# Supplementary material for: Stable and selective permeable hydrogel microcapsules for high-throughput cell cultivation and enzymatic analysis
Source: Microb Cell Fact. 2020 Aug 27;19:170. doi: 10.1186/s12934-020-01427-9 (PMC7451113; doi:10.1186/s12934-020-01427-9)
Supplement: Supplementary file 1 — Additional file 1: Figure S1. Poisson Statistics and enrichment of colonized microcapsules. a Shown is the probability that a colonized microcapsule is monoclonal according to the Poisson distribution. During cell encapsulation, the lower the average number of viable cells per capsule (λ), the higher the probability that a colonized microcapsule is monoclonal (red). A low average number of cells per capsule leads to a lower fraction of monoclonal microcapsules (blue) and, in turn, to a higher fraction of uncolonized microcapsules. b Scheme of the gravity-driven separation procedure for the enrichment of colonized microcapsules from a mixture of colonized and uncolonized microcapsules. Figure S2. Sortase enzymes catalyze a transpeptidation reaction. a Scheme of the transpeptidation reaction catalyzed by sortase enzymes. Sortases recognize the pentapeptide sorting motif (e.g. LPXTG here) on the target protein (acyl donor substrate); The cysteine in the active site of the enzyme performs a nucleophilic attack on the carbonyl carbon of threonine residue, breaking the peptide bond between the threonine and the glycine and forming an intermediate in which the enzyme and the target protein are linked together via a thioacyl linkage. The subsequent nucleophilic attack of the thioacyl linkage by the free amino group of an oligoglycine stretch of a cell wall component (acyl acceptor substrate) resolves the intermediate and results to the covalent attachment of the target protein to the bacterial cell wall. b Scheme of the intramolecular transpeptidation reaction catalyzed by an N-terminally-modified sortase enzyme. The glycine residue at the N-terminus acts as the acyl acceptor substrate that solves the intermediate in an intramolecular fashion. This enables the sortase enzyme to covalently capture the acyl donor substrate (e.g. a FITC-labelled pentapeptide). [file 12934_2020_1427_MOESM1_ESM.docx]

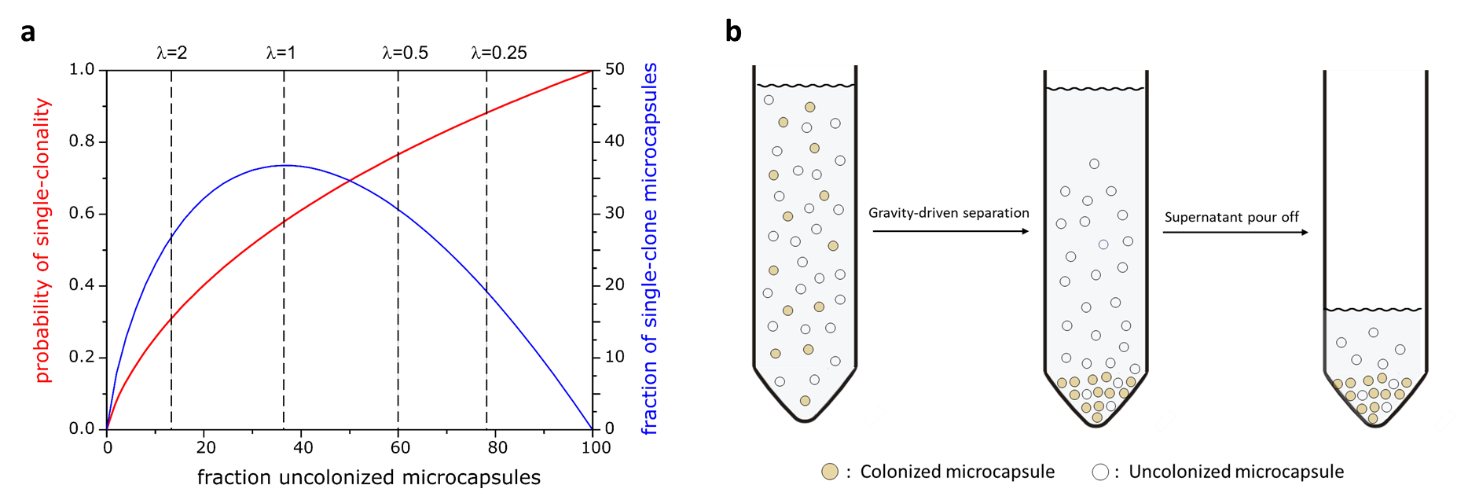


**Figure S1: Poisson Statistics** **and enrichment of colonized microcapsules**. **a** Shown is the probability that a colonized microcapsule is monoclonal according to the Poisson distribution. During cell encapsulation, the lower the average number of viable cells per capsule (λ), the higher the probability that a colonized microcapsule is monoclonal (red). A low average number of cells per capsule leads to a lower fraction of monoclonal microcapsules (blue) and, in turn, to a higher fraction of uncolonized microcapsules. **b** Scheme of the gravity-driven separation procedure for the enrichment of colonized microcapsules from a mixture of colonized and uncolonized microcapsules.


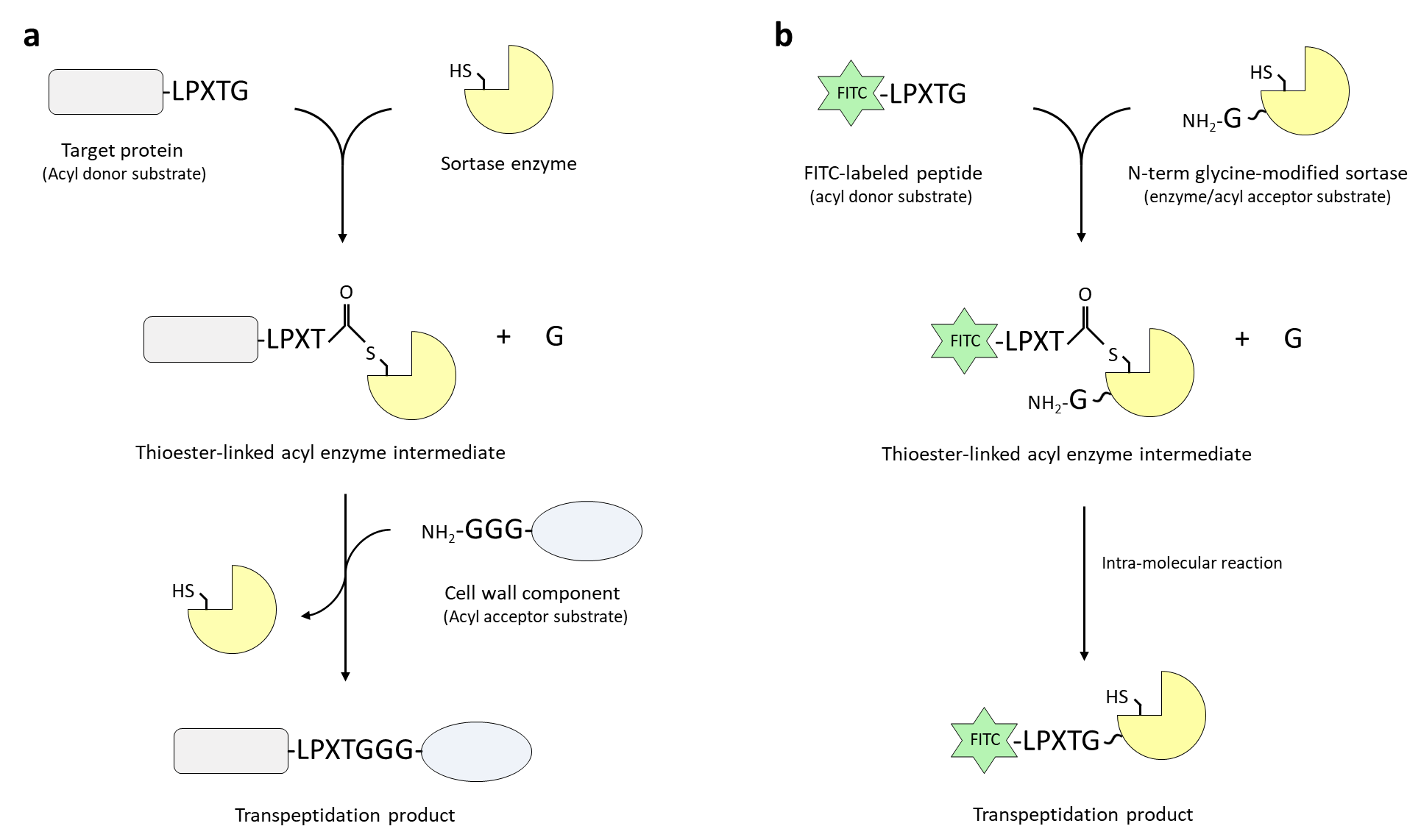


**Figure S2: Sortase enzymes catalyze a transpeptidation reaction. a** Scheme of the transpeptidation reaction catalyzed by sortase enzymes. Sortases recognize the pentapeptide sorting motif (e.g. LPXTG here) on the target protein (acyl donor substrate); The cysteine in the active site of the enzyme performs a nucleophilic attack on the carbonyl carbon of threonine residue, breaking the peptide bond between the threonine and the glycine and forming an intermediate in which the enzyme and the target protein are linked together via a thioacyl linkage. The subsequent nucleophilic attack of the thioacyl linkage by the free amino group of an oligoglycine stretch of a cell wall component (acyl acceptor substrate) resolves the intermediate and results to the covalent attachment of the target protein to the bacterial cell wall. **b** Scheme of the intramolecular transpeptidation reaction catalyzed by an N-terminally-modified sortase enzyme. The glycine residue at the N-terminus acts as the acyl acceptor substrate that solves the intermediate in an intramolecular fashion. This enables the sortase enzyme to covalently capture the acyl donor substrate (e.g. a FITC-labelled pentapeptide).
